# Factors associated with adherence to oral HIV pre-exposure prophylaxis among female sex workers in the Mara Region, Tanzania: A 2022 cross-sectional study

**DOI:** 10.1097/MD.0000000000034824

**Published:** 2023-09-08

**Authors:** Mwanaidi Churu, Khamis Kulemba, Anthony Kapesa, Alphaxard Kajura, Diana Wilfred, Namanya Basinda, Samwel Kaluvya, Kihulya Pastory Mageda

**Affiliations:** a Catholic University of Health and Allied Sciences, School of Public Health, Mwanza, Tanzania; b President’s Office, Regional Administration and Local Government, Dodoma, Tanzania; c Bugando Teaching and Consultant Hospital, Department of Obstetrics and Gynecology, Mwanza Tanzania.

**Keywords:** adherence, female sex workers, human immunodeficiency virus, pre-exposure prophylaxis

## Abstract

Female sex workers (FSWs) have a higher risk of acquiring human immunodeficiency virus (HIV) infection compared with the general female population. Tanzania adopted pre-exposure prophylaxis (PrEP) use for HIV-negative key populations in 2019 as a strategy to reduce HIV transmission. We aimed to identify factors associated with PrEP adherence among FSWs in Tanzania. We conducted a cross-sectional study of FSWs using oral HIV PrEP from June-July 2022 in 5 Mara Region districts. Stata software was used to analyze the quantitative data. Poor overall PrEP adherence was observed among FSWs, with adherence of 48.3% (174/360) and 43.3% (156/360) according to pill count and self-reports, respectively. Participants living with family members had 2.32 higher odds of PrEP adherence (adjusted odds ratio [aOR] = 2.32, 95% confidence interval [CI] = 1.27–42.23, *P* = .006) versus participants living alone or with friends. Moreover, FSWs who preferred pill packages had 1.41 higher odds of adherence (aOR = 2.43, [95% CI = 1.41–4.19, *P* = .001]); participants with a good perception of PrEP had 1.71 higher odds (aOR = 1.71, [95% CI = 1.01–2.91, *P* = .047]) of PrEP adherence. We found that living with family, pill packaging, and PrEP perception played significant roles in PrEP adherence among FSWs. Interventions to improve pill packaging, social support, and the perception of PrEP among FSWs should be intensified to increase adherence in this vulnerable population.

## 1. Introduction

Human immunodeficiency virus (HIV) infection is a global public health concern and a significant cause of death, especially in developing countries.^[1,2]^ Sub-Saharan Africa is the most severely affected region globally, accounting for nearly two-thirds of the global HIV burden. Biological, social, cultural, behavioral, economic, and structural variables contribute to this high burden.^[3]^

Estimates show that Tanzania has 1.9 million people living with HIV, of whom 77,000 contracted the disease initially and 24,000 died from acquired immunodeficiency syndrome (AIDS)-related illnesses in 2019.^[1]^ The United Nations Program on HIV/AIDS (UNAIDs) reported that the rate of new infections in Tanzania was slowly decreasing.^[4]^ The overall HIV prevalence among adults aged 15 to 49 years decreased with time, from 7% in 2003/2004 to 5.1% in 2011/2012 and 4.7% in 2016/2017.^[5]^ Studies conducted among key populations in selected regions of Tanzania reported a prevalence of 28% among female sex workers (FSWs).^[6]^ Owing to economic insecurity, inability to negotiate regular condom use, being assaulted, criminalization, and marginalization, sex workers are 14 times more likely to contract HIV than the general female population.^[7]^ Therefore, FSWs play a crucial role in the transmission of HIV.^[8]^

The World Health Organization (WHO) recommends the adoption of a number of combined preventive strategies, including anti-retroviral therapy usage among HIV-negative key populations, to reduce the number of new HIV infections.^[9]^ This strategy is generally referred to as pre-exposure prophylaxis (PrEP), and is advised by the UNAIDS and WHO for groups with an annual risk of HIV acquisition > 3%.^[10,11]^ Evidence demonstrates that PrEP is highly effective and virtually eliminates the risk of contracting HIV through sexual intercourse by 99% when used consistently and correctly.^[12]^ Tanzania adopted the use of PrEP between 2018 and 2019 to protect FSWs.^[13]^

However, despite the high risk of HIV acquisition among FSWs, most studies on PrEP adherence among FSWs have revealed minimal compliance with the provided prophylaxis.^[14–17]^ Literature on adherence to HIV oral PrEP is limited. This study aims to determine adherence and factors associated with non-adherence to oral HIV PrEP by gathering information related to the extent to which FSWs receive the PrEP service in the Mara region.

## 2. Materials and methods

### 2.1. Ethical considerations

This study was approved by the Catholic University of Health and Allied Sciences and the National Health Research Ethics Subcommittee of the National Institute for Medical Research. Participation was voluntary, and written consent was obtained from the Participants. The participants retained the right to withdraw from the study at any time.

### 2.2. Study design and setting

This cross-sectional study was conducted among FSWs in selected health facilities in the Mara Region that offered PrEP services. Five councils, namely the Tarime District Council, Tarime Town Council, Rorya District Council, Musoma Municipal Council, and Bunda Town Council, were included in the study. The Mara Region is a focus area of the National AIDS Control Programme. Through the National AIDS Control Programme, FSWs receive PrEP at clinics in accordance with the Tanzania Ministry of Health with the aim of reducing HIV transmission.

### 2.3. Study participants

The study population consisted of FSWs aged ≥ 18 years in accordance with the national guidelines for the provision of PrEP for the prevention of HIV infection in vulnerable populations.

#### 2.3.1. Sample size calculation.

The sample size of FSWs included in the present study to determine adherence to oral HIV PrEP was calculated based on the level of adherence to oral HIV PrEP among FSWs in Kampala, Uganda, which was 71%.^[18]^ The sample size was estimated using the Kish–Leslie formula (1965) as follows:^[19]^

$$\begin{array}{l} Sample\;Size\;\;\;N=Z^{2}P\frac{\left(1-P\right)}{d^{2}} \end{array}$$ (1)

where **N** = minimum sample size; **Z** = confidence interval (CI) level at 95% (CI = 1.96); **P** is the proportion of PrEP adherence among FSWs based on a previous study conducted in Kampala, Uganda 71%^[18]^; and **d** = 5% margin of error (standard value = 0.05).

The sample size was calculated as follows:

$$\begin{array}{l} N=1.96^{2}\times 0.71\frac{\left(1-0.71\right)}{0.05^{2}}=316.7 \end{array}$$ (2)

The minimum calculated sample size was 317. After adding 10% of the non-respondents the sample size was calculated as follows:

$$\begin{array}{l} \begin{array}{lll} & 10/100\;\times \;317=\;31.7\; & 31.7+317=348.9\; \end{array} \end{array}$$ (3)

Therefore, the sample size required to determine the level of adherence to the study was 349 FSWs taking oral HIV PrEP.

### 2.4. Sampling

Simple random sampling was used to select the 5 of 9 districts to be included in the study. These districts have many gold mining sites and numerous recreational sites, such as clubs, bars, hotels, pubs, massage parlors, and guesthouses, where FSWs work. To select the study participants, a simple random sampling method was used to select the health facilities that offered PrEP services for inclusion in the study, and interviews guided by community health workers familiar to the participants were conducted at the workplace. FSWs taking PrEP were proportionately divided among the selected health facilities, and a convenient sampling method was used to obtain eligible FSWs until the sample size was reached. In addition, every FSW who attended a clinic for PrEP refills or received PrEP service at the community outreach center and met the selection criteria were asked to participate in the study.

### 2.5. Variable and measure

The dependent variable was adherence, which was assessed using both self-reporting and pill count methods. The self-reporting method was used to measure adherence using 4 adherence questions, which were designed to measure adherence in a resource-constrained setting.^[20]^ In order to maximize the precision of adherence measurement, the pill count method was also deployed to measure adherence. Key and vulnerable population client cards were used to verify the number of pills dispensed during the last visit, and adherence was calculated using the following formula^[21]^:

$$\begin{array}{l} \%\;of\;missing\;pill\frac{no.of\;pills\;remain}{no.of\;pill\;dispensed}\times \;100 \end{array}$$ (4)

$$\begin{array}{l} \%\;of\;adherence=100-\%\;of\;missing\;pills \end{array}$$ (5)

Therefore, participants with > 95% pill use were considered to have good adherence.

The **Independent** variables were the number of clients per day, PrEP duration, pill color, pill package, pill refill site, education level, marital status, age, condom use, and risk level (7 questions were asked and risk was categorized by the scores). A score of 0 was regarded as no risk, 1 to 2 as low risk, 3 to 4 as moderate risk, and 5 to 7 as high risk.

### 2.6. Data collection

A structured questionnaire guided by the conceptual framework, which was developed from the social-ecological model, was used to collect data. Well-trained research assistants (healthcare workers, such as nurses) collected the data.

A structured questionnaire guided by the conceptual framework was used to determine the level of adherence and explore the factors associated with oral HIV PrEP among FSWs. Participants were recruited when refilling their prescriptions at their respective health facilities and community outreach centers. This process was facilitated by the existing healthcare workers throughout the study period. Moreover, community outreach workers helped identify eligible participants for inclusion in the study.

### 2.7. Data analysis

Stata software (StataCorp LLC, College Station, TX) was used for data analysis. Categorical variables are described as proportions or percentages. Numerical variables are presented as means and medians with their corresponding standard deviations.

Multiple logistic regression analysis was performed to examine the association between the independent variables and adherence. The crude association of each independent variable was determined to examine its relationship with the dependent variable (adherence) in univariate models. Any variable with a *P*-value < 0.10 in the univariate test was considered a candidate for the multivariable model along with the variables of known clinical importance. Once the variables were identified, they were entered into the multivariate model. Associations are presented as odds ratios with 95% CIs.

We used the Hosmer-Lemeshow test to examine whether the final model adequately fitted the data for the multiple logistic regression model. An interaction test was performed to examine the effects of heterogeneity. The final parsimonious model (i.e., the model with significant findings for predictors) is presented. The model-building procedure and guidelines for reporting the regression analysis are described in detail elsewhere.

## 3. Results

### 3.1. Sociodemographic characteristics of study participants

A total of 360 FSWs taking PrEP were enrolled in this study from June to July 2022. More than half of the participants (299/360, 83.1%) were Christians; 65% were older than 25 years; and the mean age was 28 ± 7 years. Most participants (225/360, 62.5%) were single, and almost half (179/360, 49.7%) had attained a primary level of education. Approximately one-third of participants (121/360, 33.6%) reported sex work as their main source of income. Among the 239 FSWs who reported that they had other jobs besides sex work, the majority (149/360, 62.3%) worked as bartenders. Details of the results are presented in Table 1.

**Table 1:** Social demographic characteristics of the FSWs in Mara, June 2022 (N = 360).

Variable	Number	Proportion
Place of interview		
Health facility	47	13.1
Community centre	313	86.9
Age		
15–19	30	8.3
20–24	96	26.7
25+	234	65
Trible		
Kurya	111	30.8
Jita	75	20.8
Luo	38	10.6
Zanaki	47	13.1
Others	89	24.7
Residence and duration		
Mara >1 yr	322	89.4
Mara <1 yr	38	10.6
Religion		
Christian	299	83.1
Muslim	61	16.9
Marital status		
Single	61	16.9
Married	225	62.5
Divorced/Separated	4	1.1
Education level		
No formal education	12	3.3
Primary education	179	49.7
Secondary and above	169	46.9
Living arrangement		
Alone	83	23.1
family	115	31.9
Friends	161	44.7
Is sex work your main source of income		
Yes	121	33.6
No	239	66.4
Other works		
Bartender	149	62.3
Catering	33	13.8
Work in barbershop	21	8.8
Other occupations	36	15.1
Currently, take/drink alcohol		
Yes	335	93.1
No	25	6.9
Council		
Tarime DC	41	11.4
Tarime TC	112	31.1
Rorya DC	21	5.8
Musoma MC	141	39.2
Bunda TC	45	12.5
Tarime DC	41	11.4

FSW = female sex worker.

### 3.2. HIV acquisition risk assessment among FSWs in mara region

The results showed that 54.2% (195/360) of participants had been working as a sex worker for more than 3 years. More than half of the participants (258/360, 71.7%) began engaging in commercial sex work when they were ≥ 18 years old, and approximately a quarter of FSWs (103/360, 28.6%) claimed to have at least 3 sex clients per day; 84.7% (305/360) used condoms for HIV prevention, although 80% (288/360) reported inconsistent condom use. Regarding HIV risk assessment, a relatively large number of participants (245/360, 68.1%) had moderate risk (Table 2).

**Table 2:** History of being at risk of contracting HIV among FSWs in Mara, June 2022 (N = 360).

Variable	Number	Proportion
Duration working as a sex worker		
≥3 yr	195	54.2
<3 yr	165	45.8
Age start commercial sex work		
<18 yr	102	28.3
≥18 yr	258	71.7
Number of sexual clients per d		
≥3 sexual clients	103	28.6
<3 sexual clients	257	71.4
Participant use condoms for HIV prevention		
Yes	305	84.7
No	55	15.3
Participant use condoms consistently		
Yes	17	4.7
No	288	80.0
Using lubricant during last sexual encounter		
Yes	47	13.1
No	313	86.9
Participant use injectable drugs		
Yes	7	1.9
No	353	98.1
Participant level of risk of acquiring HIV		
High risk	89	24.7
Moderate risk	245	68.1
Low risk	26	7.2

FSW = female sex worker, HIV = human immunodeficiency virus.

### 3.3. PrEP characteristics and preferences of FSWs

All participants reported being counseled and screened for HIV infection and undergoing other baseline examinations before PrEP initiation. During the study period, all participants (360/360, 100%) received PrEP, although the duration of PrEP use varied. Approximately half (184/360, 51.1%) of the FSWs reported using PrEP for 4 to 6 months; however, 81.4% (293/360) disliked the pill size. Watch and phone reminder applications were used to remind participants to take pills by 89.7% (323/360) and 10.3% (37/360) of participants, respectively. The majority of participants (215, 59.7%) were willing to join a PrEP adherence support group, and 61.7% (222/360) suggested injectable as the preferred alternative PrEP formulation (Table 3).

**Table 3:** PrEP adherence characteristics among FSWs in Mara region, June 2022 (N = 360).

Variable	Number	Proportion
Counseled before PrEP initiation		
Yes	360	100.0
No	0	0.00
Screened for HIV and other important investigation before PrEP initiation		
Yes	360	100.0
No	0	0.00
Duration on PrEP		
<3 mo	89	24.7
4–6 mo	184	51.1
>6 mo	87	24.2
Like the pill color		
Yes	298	82.8
No	62	17.2
Like the pill size		
Yes	67	18.6
No	293	81.4
Like the current pill packaging		
Yes	151	41.9
No	209	58.1
Reminders to take PrEP		
App	37	10.3
Watch	323	89.7
PrEP refill sites		
Health facilities	170	47.2
Community based	190	52.8
Participant taking other medication rather than PrEP		
Yes	28	7.8
No	332	92.2
Can you afford PrEP if it not free?		
Yes	38	10.6
No	322	89.4
How do health care workers treat you a being female sex worker		
Good	32	8.9
Fair	312	86.7
Poor	16	4.4
Would you like to join a PrEP adherence group?		
Yes	215	59.7
No	145	40.3
Would you like to receive message reminders about PrEP?		
Yes	331	91.9
No	29	8.1
Would you like to receive message reminders about PrEP?		
Yes	331	91.9
No	29	8.1
Other PrEP formulation suggest by participant		
Injectable	222	61.7
Syrup	61	16.9
Gel	77	21.4

HIV = human immunodeficiency virus, PrEP = pre-exposure prophylaxis.

### 3.4. PrEP adherence assessment

**Figure 1.**
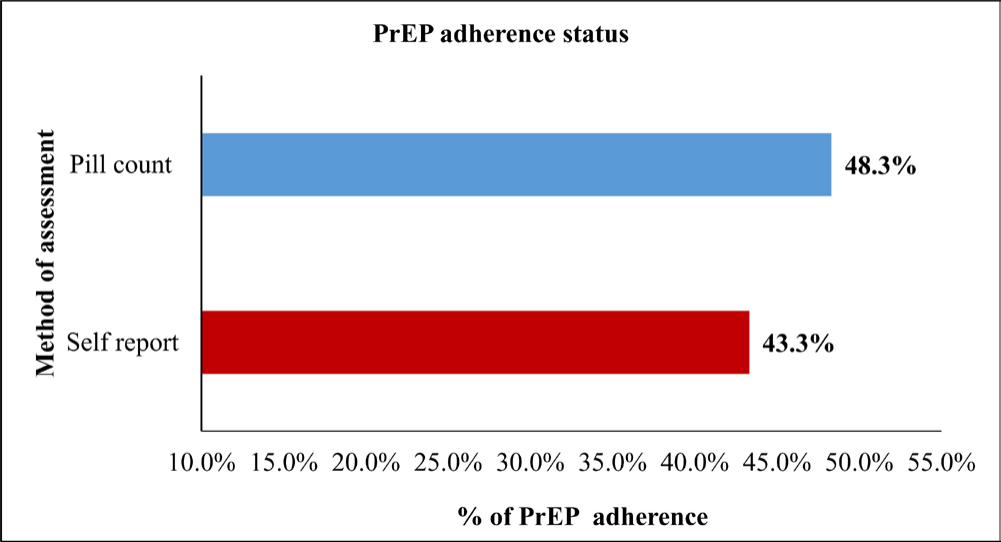
Result of pre-exposure prophylaxis adherence according to self-report and pill count.

Generally, PrEP adherence was poor among participants in this study, with only 48.3% (174/360) and 43.3% (156/360) of participants adhering according to pill count and self-reporting, respectively. The agreement measure between the pill count and self-report methods was moderate, with kappa statistics of 0.44 (*P* < 0001) (Fig. 1).

### 3.5. Factors that hinder PrEP adherence among FSWs

More than half of the participants (209, 58.1%) encountered challenges while accessing PrEP. The commonly reported challenges were stigma (47, 13.1%), lack of privacy when accessing pills (10, 2.8%), distance from health facilities (50, 13.9%), delayed service delivery (67, 18.6%), and work overload (35, 9.7%). In contrast, 238 (66.1%) participants experienced challenges while taking PrEP, with the most common being forgetfulness (106, 29.4%) and side effects (98, 27.2%) (Table 4).

**Table 4:** Factors that hindered PrEP adherence among FSWs in June 2022.

Variable	Number	Proportion
Do you encounter challenges with accessing PrEP?		
Yes	209	58.1
No	151	41.9
Challenges encountered with accessing PrEP		
Stigma	47	13.1
Lack of privacy when accessing pills	10	2.8
Distance of health facilities	50	13.9
Delayed service delivery	67	18.6
Work overload	35	9.7
Do you encounter challenges while taking PrEP?		
Yes	235	65.3
No	125	34.7
Challenges encountered while taking PrEP		
Pill burden	5	1.4
Stigma	16	4.4
Forgetfulness	106	29.4
Pill size	5	1.4
Side effects	98	27.2
Need to hide when taking pills	6	1.7
Travel	3	0.8

PrEP = pre-exposure prophylaxis.

### 3.6. Factors associated with good adherence to oral HIV PrEP among FSWs

In the multivariable analysis, living with family members adjusted odd ratio (aOR = 2.32, [95% CI = 1.27–4.23; *P* = .006]) was an independent predictor of good adherence; participants living with family members were twice as likely to adhere to PrEP than participants living alone. The second predictor of good adherence was pills packaging. Participants who reportedly loved the current pill packaging had 2.43 higher odds of PrEP adherence (aOR = 2.43, [95% CI = 1.41–4.19; *P* = .001]) compared with participants who disliked the pill packaging. The final predictor of good adherence was perception; participants who had a good perception of PrEP had 1.01 higher odds of PrEP adherence (aOR = 1.71 [95% CI = 1.01–2.91; *P* = .047]) compared with participants with a poor perception of PrEP (Table 5). The remaining factors examined as independent predictors of adherence showed no significant effects.

**Table 5:** Bivariate and multivariate analysis of factors associated with adherence of oral HIV pre-exposure prophylaxis.

Variable	Adherence status		Bivariate		Multivariate	
	Good (n)	Poor (n)	cOR (95% CI)	*P* value*	aOR (95% CI)	*P* value
Age						
25 and above	117	117	1			
20–24	40	56	0.714 (0.442–1.154)	.169		
15–19	16	14	1.14 (0.534–2.448)	.743		
Religion of the participants						
Muslim	144	156	1			
Christian	29	31	1.01 (0.58–1.77)	.962		
Marital status of the participants						
Divorced/Separated	70	61	1			
Single	101	124	1.41 (0.914–2.17)	.120		
Married	2	2	1.15 (0.16–8.39)	.892		
Highest Educational level attained						
Secondary education and above	69	100	1			
No formal education	6	6	1.45 (0.45–4.68)	.535	1.24 (0.31–4.98)	.758
Primary education	99	80	1.79 (1.17–2.74)	**.007**	1.26 (0.74–2.17)	.398
Is sex main source of income						
No	114	125	1			
Yes	60	61	1.08 (0.69–1.67)	.735		
Currently taking/drinking alcohol						
No	13	12	1			
Yes	161	174	0.854 (0.38–1.93)	.704		
Living arrangement						
Friends	64	97	1			
Alone	40	43	1.41 (0.827–2.405)	.207	1.13 (0.58–2.18)	.725
family	69	46	2.27 (1.395–3.706)	**.001**	2.32 (1.27–4.23)	**.006**
Duration working as a sex worker						
<3 yr	71	92	1			
≥3 yr	103	94	1.48 (0.97–2.25)	**.064**	0.81 (0.46–1.44)	.479
Age start commercial sex work						
≥18 yr	127	131	1			
<18 yr	47	55	0.88 (0.56–1.39)	.803		
Number of sexual clients per day						
<3 sexual clients	117	140	1			
≥3 sexual clients	63	40	2.07 (1.29–3.30)	**.002**	1.81 (0.89–3.69)	.100
Use of condoms consistency for HIV Prevention						
No	133	155	1			
Yes	12	5	2.79 (0.96–8.14)	.059	1.69 (0.32–8.91)	.531
Duration on PrEP						
>6 mo	46	41	1			
4–6 mo	86	98	0.78 (0.47–1.30)	.346		
<3 mo	42	47	0.79 (0.44–1.44)	.451		
Color of the pill						
No	123	170	1			
Yes	51	16	4.41 (2.39–8.09)	**<.001**	0.97 (0.49–1.93)	.929
Pill package						
No	75	134	1			
Yes	99	52	3.40 (2.19–5.28)	**<.001**	2.43 (1.41–4.19)	**.001**
Reminders used to take pill						
Watch	148	175	1			
App on phone	26	11	2.79 (1.34–5.85)	**.006**	0.53 (0.21–1.31)	.166
Level of risk of acquiring HIV						
Low risk (score 1–2)	16	10	1			
Moderate risk (score 3–4)	107	138	0.83 (0.34–2.05)	**.087**	0.54 (0.14–2.10)	.372
High risk (score 5–7)	51	38	0.48 (0.211–1.11)	.700	0.66 (0.14–3.02)	.590
Area of drug refill						
Health facility	72	98	1			
community	102	88	1.58 (1.04–2.394)	**.032**	0.94 (0.55–1.63)	.83
Health care provider treat you a being FSW						
Poor	8	8	1			
fair	153	159	0.96 (0.35–2.63)	.940		
Good	13	19	0.68 (0.21–2.29)	.538		
Perception on PrEP						
Poor	70	111	1			
Good	104	75	2.19 (1.44–2.35)	**<.001**	1.71 (1.01–2.91)	**.047**

aOR = adjusted odds ratio, CI = confidence interval, cOR = crude odds ratio, FSW = female sex worker, HIV = human immunodeficiency virus, PrEP = pre-exposure prophylaxis.

*χ ^2^ *P* value.

## 4. Discussion

To reduce the number of new HIV infections, the WHO recommends the use of several prevention interventions combined, including ARV, among HIV-negative individuals with a substantial risk of HIV infection. This primary preventive approach is known as PrEP. Evidence shows that when taken consistently and correctly, PrEP is very effective and reduces the risk of HIV infection to near zero. The present study focused on identifying the factors associated with adherence to PrEP among FSWs. Our study found that the overall PrEP adherence among FSWs was 48.3%, which was lower than that observed in other countries.^[18,22]^ The present study found that living with family, pill packaging, and perception of PrEP were associated with adherence.

This study provides evidence that living arrangements, such as living with family, are associated with good adherence to PrEP. The findings indicate that when a sex worker is a member of a family and the family depends on her self-prevention of acquiring HIV, she can continue to provide for her family through sex work. This further indicates that families can provide adherence support to participants. Our study findings were similar to those reported by Gombe et al^[23]^ and Jackson-Gibson et al,^[3]^ which showed that living arrangements positively improved adherence to PrEP. Our results indicate that when FSWs disclose their work to a family member, they show enhanced adherence to HIV prevention strategies. Hence, strengthening the disclosure of sex work as a source of income to families should be emphasized during PrEP education in this population.

Our data provides evidence that pill packaging is associated with good adherence among this population. This finding indicates that packaging, including colors, may influence adherence to PrEP. This finding is similar to that reported in another study by Gombe et al,^[21]^ which showed that pill packaging is important for improving adherence to PrEP medication. Our findings indicate that pill packaging should be enhanced to promote adherence to PrEP and other related medications.

This study further demonstrated that a good perception of PrEP was associated with good adherence, indicating that sex workers with a good perception of PrEP adhered to PrEP. According to the PrEP implementation framework, all vulnerable people receive education and are assessed for eligibility before initiating this medication. Thus, sex workers who are well educated before receiving PrEP should have a positive perception, which enhances their adherence. Our findings are similar to those of another study conducted in Zimbabwe by Gombe et al,^[22]^ which showed that good adherence was associated with the perception of the medication given. We recommend strengthening appropriate information on PrEP among sex workers to enhance adherence in this population.

## 5. Limitation

This study adopted a cross-sectional study design without a control group; thus, further studies are needed to show the temporal relationship. Moreover, our cross-sectional design did not include sex workers lost to follow-up. Because these sex workers were more likely to have experienced poor adherence, the results were biased toward better adherence by (naturally) excluding them.

## Acknowledgments

The authors acknowledge the school of Public Health at Catholic University of Health and Allied Sciences, as well as all community outreach volunteers under Amref-Afya Kamilifu in the Mara region for participating in data collection in collaboration with the research participants from the Musoma MC, Bunda TC, Tarime TC, Tarime DC, and Rorya DC.

## Author contributions

**Conceptualization:** Mwanaid Churu, Alphaxard Kajura, Anthony Kapesa, Namanya Basinda.

**Data curation:** Mwanaid Churu, Khamis Kulemba.

**Formal analysis:** Mwanaid Churu, Diana Wilfred, Samwel Kaluvya.

**Methodology:** Mwanaid Churu, Alphaxard Kajura, Anthony Kapesa, Kihulya Pastory Mageda.

**Project administration:** Mwanaid Churu.

**Supervision:** Alphaxard Kajura, Anthony Kapesa, Diana Wilfred, Samwel Kaluvya, Namanya Basinda.

**Writing – original draft:** Mwanaid Churu.

**Writing – review & editing:** Kihulya Pastory Mageda.
